# Effect of the PRECEDE-PROCEED model on health programs: a systematic review and meta-analysis

**DOI:** 10.1186/s13643-022-02092-2

**Published:** 2022-10-09

**Authors:** Junghee Kim, Jaeun Jang, Bora Kim, Kyung Hee Lee

**Affiliations:** 1 Department of Nursing, Yonsei University Wonju College of Nursing, Wonju, 26426 South Korea; 2 Yonsei University College of Nursing, Seoul, 03722 South Korea; 3 College of Nursing and Brain Korea 21 FOUR Project, Yonsei University, Seoul, 03722 South Korea; 4 Yonsei University College of Nursing and Mo-Im Kim Nursing Research Institute, 50-1 Yonsei-Ro, Seodaemun-Gu, Seoul, 03722 South Korea

**Keywords:** Education, Health behavior, Health promotion, Knowledge, PRECEDE-PROCEED model

## Abstract

**Background:**

The predisposing, reinforcing, and enabling constructs in educational diagnosis and evaluation policy, regulatory, and organizational constructs in educational and environmental development (PRECEDE-PROCEED) model has been used as a theoretical framework to guide health promotion strategies to prevent chronic diseases and improve the quality of life. However, there is a lack of evidence as to whether applying the PRECEDE-PROCEED model effectively improves health outcomes. This study aimed to systematically review intervention studies that applied the PRECEDE-PROCEED model and examine its effectiveness.

**Methods:**

In December 2020, seven databases were systematically searched. The quality of studies was assessed using the Mixed Methods Appraisal Tool. The outcome of interest for the meta-analysis was knowledge.

**Results:**

In total, 26 studies were systematically reviewed. Most studies provided educational programs as the main intervention for various population groups. Symptom or disease management and health-related behavior promotion were the most common topics, and education was the most frequently used intervention method. The PRECEDE-PROCEED model was applied in the planning, implementation, and evaluation of the intervention programs. Eleven studies were included in the meta-analysis, which showed that interventions using the PRECEDE-PROCEED model significantly improved knowledge.

**Conclusions:**

This study indicated that individuals are more likely to engage in health-related behaviors with better knowledge. Thus, the PRECEDE-PROCEED model can be used as the theoretical framework for health promotion interventions across population groups, and these interventions are particularly effective with regard to knowledge improvement.

## Background

The prevalence of chronic diseases is expected to increase continuously worldwide, and this phenomenon is closely related to lifestyle habits such as smoking, drinking, physical inactivity, and unhealthy eating habits [1]. Proper health promotion plans and strategies are necessary to prevent chronic diseases and improve quality of life throughout life [2]. Health promotion strategies should be employed by individuals, organizations, and communities as health goals can be accomplished through mutual efforts between participants and healthcare professionals [1]. When healthcare professionals implement health promotion programs, they are able to identify the health risk or enrichment factors of participants. Theory-based health promotion programs are important to evaluate whether these factors are appropriate [3].

The Predisposing, Reinforcing, and Enabling Constructs in Educational Diagnosis and Evaluation-Policy, Regulatory, and Organizational Constructs in Educational and Environmental Development (PRECEDE-PROCEED) model is a useful theoretical framework for planning, conducting, and evaluating health promotion programs [4]. It has been used to promote health programs for more than 30 years [3]. Green [5] developed the PRECEDE section for health diagnosis and education needs and later added the PROCEED section to the framework by adding elements of policy, regulation, organization, and the environment to emphasize the effects of ecological aspects in the modified model [4]. The model consists of eight phases: social assessment, epidemiological assessment, educational/ecological assessment, administrative/policy assessment and intervention planning, implementation, process evaluation, impact evaluation, and performance evaluation. It is a multistep approach for the development and implementation of a health promotion program [4].

A considerable number of studies have used the PRECEDE-PROCEED model primarily in the planning, implementation, and evaluation stages of various health promotion programs across populations and settings [6–9]. A study on prevention education for high-risk diabetic patients reported that eating habits effectively promoted preventive behaviors [7]. According to a previous study that assessed school safety education programs for elementary school students, the safety program impacted safety knowledge and performance ability [6]. In addition, health-related quality of life was found to improve with the quality of life education program using the PRECEDE-PROCEED model for women-headed households [8]. However, whether the PRECEDE-PROCEED model was effective across the studies remains unknown. Although the use of the PRECEDE-PROCEED model has been recommended, several studies have used only the PRECEDE section of the model as their theoretical framework [10–14]. Therefore, this study aimed to systemically analyze previous studies that used the PRECEDE-PROCEED model. A meta-analysis was also performed to examine the effectiveness and usefulness of health promotion intervention across different settings and populations.

## Methods

This study was performed according to the Preferred Reporting Items for Systematic reviews and Meta-Analyses (PRISMA) Protocols [15]. This protocol has been registered on INPLASY (registration number: INPLASY202250017; https://inplasy.com/inplasy-2022-5-0017).

### Eligibility criteria

The eligibility criteria for the studies were as follows: (a) studies containing participants of all ages, healthy people, and people with diseases in the community and hospital settings; (b) intervention studies (i.e., randomized controlled trial, quasi-experimental study) that used the PRECEDE-PROCEED model, excluding those that used only the PRECEDE model and observational studies; and (c) studies containing health-related outcomes, with behavior, cognitive and physiological health, and quality of life as primary outcomes, as well as other predisposing factors for effective intervention based on the PRECEDE-PROCEED model.

### Search strategy

Seven electronic databases were used to search for relevant studies. The databases used to search for the published articles were PubMed, the Cumulative Index to Nursing and Allied Health Literature (CINAHL), PsycINFO, and Scopus; the databases used to search for the gray literature were ProQuest, Regional Information Sharing Systems (RISS), and OpenGrey. To include studies using the PRECEDE-PROCEED model as a theoretical framework, the following search term combinations were used: [Precede-Proceed AND Model] OR [Precede-Proceed Model OR Precede Proceed] OR [Precede-Proceed AND health promotion] OR [Precede-Proceed AND community health planning] OR [Precede-Proceed AND population-based planning] OR [Precede-Proceed AND health program] OR [Precede-Proceed AND program evaluation] OR [Precede-Proceed AND intervention]. The language was limited to English, and the publication date was not restricted. The final search was conducted in December 2020.

### Data extraction

We reviewed all the titles and abstracts of the retrieved studies and removed duplicates. Two reviewers (JK and BK) determined the inclusion criteria and reviewed the full text of the selected articles. If their decision was unanimous, a third reviewer (KL) confirmed the inclusion criteria. Three independent reviewers (JK, BK, and KL) used a custom form developed to extract the appropriate data. The following data were extracted: study country, study design, participants, number and mean age of the intervention and control groups, duration and frequency of intervention, outcomes, and application of the PRECEDE-PROCEED model.

### Study quality assessment

Quality assessment was performed independently by two reviewers (JK and BK) using the Mixed Methods Appraisal Tool (MMAT), which systematizes the critical evaluation of non-randomized and randomized quantitative studies [16]. Disagreement was resolved by independent assessment by a third reviewer (KL). The overall quality scores of this tool are a combination of quality criteria designed to assess the appropriateness of sampling, measurement, rates of data completion, and the number of factors aimed to determine the potential for bias. Two screening questions were utilized to determine whether there was a clear research question and if the collected data addressed the research questions. Subsequently, non-randomized and randomized quantitative studies were evaluated using five questions with “Yes,” “No,” and “Can’t tell” answers. After evaluation according to each criterion, the number of “yes” responses was rated.

### Data synthesis and analysis

We summarized the publication year, country, setting, study design, participants’ characteristics, intervention, duration of session, outcome, and application of the PRECEDE-PROCEED model.

For the meta-analysis of the primary outcome (i.e., knowledge) among the outcome values, we entered the data into the Comprehensive Meta-Analysis (CMA) version 3.3 program based on each study design and outcome measurement (number of participants, mean, standard deviation, and *p* value). The outcomes of the study were addressed using the standardized mean difference (SMD) to report the standardized mean difference of the continuous outcome type data and 95% confidence interval (CI), as well as the *p* values of the SMDs. We used a random effects model to reduce the effect of statistical heterogeneity on the evaluation and reported the *I*^2^ statistic.

## Results

### Study selection

**Fig. 1:**
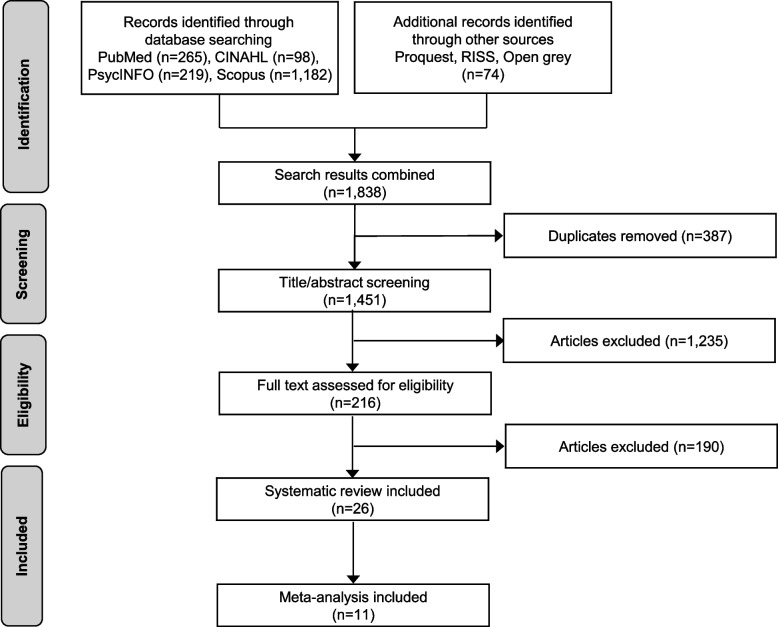
Flow diagram of the study selection

The initial searches identified 1,838 records from seven electronic databases; after removing duplicates, 1,451 studies remained. Furthermore, from the review of titles and abstracts, 1,235 studies were excluded because of the study design (mixed-method studies, qualitative studies, and review papers) and because they applied the PRECEDE model only. Following the examination of the full text of articles, observational studies (*n* = 190) were excluded, and experimental studies or quasi-experimental studies (*n* = 26) were included. Of these, 15 studies were not combined in the meta-analysis; hence, only 11 studies were included in the meta-analysis (Fig. 1).

### Study characteristics

The study characteristics are presented in Table 1. All included studies were published after 2000: 7 studies (26.9%) between 2003 and 2010, 6 studies (23.1%) between 2011 and 2015, and 13 studies (50.0%) after 2015. Approximately half of all studies were published within the last 5 years. Among the 26 studies, nine were conducted in Iran (34.6%), six in the USA (23.1%), two in Canada (7.7%), and two in Thailand (7.7%). The remaining seven studies (26.9%) were conducted in different countries (China, Indonesia, Peru, Spain, Taiwan, Tanzania, and Turkey). Study settings included were hospitals (*n* = 12) and communities (*n* = 14) such as schools or universities (*n* = 6), health centers (*n* = 4), community pharmacies, military installations, districts, and vocational program sites. Regarding the study design, 12 studies (46.2%) were randomized controlled trials (RCTs), and 14 studies (53.8%) had a quasi-experimental research design.

**Table 1:** Characteristics of the included studies (*n* = 26)

Author	Country/setting	Design	Participants	Intervention (*n*/mean age)	Duration (frequency)^c^	Control (*n*/mean age)	Knowledge as a primary outcome	Secondary outcome	Application of the PRECEDE-PROCEED model
Adamuz et al. [17]	Spain/hospital	RCT	CAP participants	Individualized education (102/65.00^a^)	30 (2)	Conventional information (105/72.00^a^)	Yes	Additional healthcare visits and rehospitalization	Planning, implementation, evaluation
Azar et al. [18]	Iran/hospital	QE	DM participants	Self-management education (43/56.65)	45–60 (8)	Routine care, training (43/55.09)	Yes	Quality of life	Planning, implementation, evaluation
Barasheh et al. [19]	Iran/health center	QE	Type 2 DM participants	Self-care education (55/48.74)	60 (4)	Routine care and training (55/79.89)	Yes	Self-care behavior	Planning
Bazpour et al. [20]	Iran/hospital	RCT	Beta-thalassemia participants	Lifestyle education (32/18.30)	45–60 (6)	NS (32/17.84)	Yes	Lifestyle	Planning, implementation, evaluation
Bridges et al. [21]	USA/university	QE	College students	Suicide Prevention and Awareness for Depression program (329/20.54)	NS (4)			Depression awareness	Planning, implementation, evaluation
Cannick et al. [22, 23]	USA/university	RCT	Dental students	Education for oral cancer prevention and detection (52/24.35^b^)	120 (1)	Usual instruction (52/24.35^b^)	Yes	Competency	Planning, implementation, evaluation
Chabot et al. [24]	Canada/community pharmacies	QE	HTN participants	Pharmacist intervention (41/68.00)	120 (1)	NS (59/63.00)		Blood pressure	Planning, implementation, evaluation
Chiang et al. [25]	Taiwan/hospital	RCT	Parents of asthmatic children	Self-management education (33/NS)	NS (1)	Regular outpatient asthma education (35/NS)	Yes	Self-management	Planning
Cole & Horacek [26]	USA/military installation	RCT	Military spouses	Intuitive eating program (18/37.50)	60 (10)	NS (14/37.00)		Intuitive eating attitude	Planning, implementation, evaluation
Didehvar et al. [27]	Iran/hospital	QE	Nurses and midwives	Stress management training (62/30.76)	240 (2)			Job stress	Planning
Gary et al. [28]	USA/hospital	RCT	Type 2 DM participants	1. NCM and usual care (38/59.00) 2. CHW and usual care (41/59.00) 3. NCM/CHW and usual care (36/60.00)	2 years^d^	Usual care (34/57.00)		HbA1c	Planning
Kaewchin et al. [29]	Thailand/school	RCT	Overweight and obese students	Behavioral modification (40/NS)	45 (12)	NS (40/ NS)		Health literacy and behavioral modification	Planning, implementation
Kattelmann et al. [30]	USA/university	RCT	College Student	Web-delivered intervention (824/19.40)	NS (21)	Delayed intervention (815/19.30)		Weight, BMI, fruit and vegetable intake, physical activity, and perceived stress	Planning
Khortwong & Kaewkungwal [31]	Thailand/hospital	QE	TB participants	Health education (50/29.16)	20–30 (12)	Routine treatment (50/28.08)		Compliance and quality of life	Planning, implementation, evaluation
Kim et al. [32]	Peru/school	QE	Students	Mental health education (760/NS)	9 months^d^	NS (746/NS)		Mental health-related risk behaviors and suicide attempts	Planning, implementation, evaluation
Lacaille, et al. [33]	Canada/hospital	QE	IA participants	Self-management program (19/51.00)	180 (5)			Self-efficacy	Planning
Lin et al. [34]	China/hospital	QE	OR nurses	OBE management program (87)	120–180 (3)			Attitudes, and behavior for OBE prevention	Planning, implementation, evaluation
Mazloomymahmoodabad, et al. [35]	Iran/health center	QE	Older adults	Healthy behavior education (64/65.80)	9 months^d^	NS (64/67.80)	Yes	Quality of life	Planning
Moshki et al. [7]	Iran/health center	QE	High-risk participants of type 2 DM	Preventive behavior education (82/37.50)	90 (1)	NS (82/38.59)	Yes	Preventive behavior	Planning, implementation, evaluation
Moshki et al. [36]	Iran/health center	RCT	Menopausal women	Self-efficacy and self-acceptance education (40/52.60)	120 (4)	Booklet (40/50.80)	Yes	Self-efficacy and self-acceptance	Planning, implementation
Ngowi et al. [37]	Tanzania/district	RCT	Pig farmers	Infection control education (393/33.00^a^)	NS (1)	NS (434/32.00^a^)		Incidence rate of porcine cysticercosis	Planning, implementation, evaluation
Ranjbaran et al. [38]	Iran/hospital	RCT	CABG participants	Education for cardiac rehabilitation (50/59.30)	45–60 (8)	Exercise and lifestyle training (50/59.50)	Yes	Poor sleep quality	Planning
Sezgin & Esin [39]	Turkey/hospital	QE	ICU nurses	Ergonomic risk management education (35/27.00^b^)	2 weeks^d^	NS (37/27.00^b^)		Musculoskeletal symptoms	Planning, implementation, evaluation
Syakurah et al. [40]	Indonesia/university	QE	Medical students	Family health promotion education (244/NS)	5 weeks^d^			Self -assessment on activities	Planning, implementation, evaluation
Pournaghash-Tehrani & Etemadi [41]	Iran/hospital	RCT	CABG with ED participants	Sex education (55/59.30^b^)	NS (8)	Regular training (55/59.30^b^)	Yes	Quality of life	Planning, implementation, evaluation
Walsh et al. [42]	USA/vocational training program site	QE	Low-income young adults	Healthy lifestyle intervention (89/20.00^b^)	10 weeks^d^	No intervention (76/20.00^b^)		Physical activity and eating behavior	Planning, implementation, evaluation

*BMI* Body mass index, *CABG* Coronary artery bypass graft, *CAP* Community-acquired pneumonia, *CHW* Community health worker, *DM* Diabetes mellitus, *ED* Erectile dysfunction, *HTN* Hypertension, *IA* Inflammatory arthritis, *ICU* Intensive care unit, *NCM* Nurse case manager, *NS* Not specified, *OBE* Occupational blood-borne pathogen exposure, *OR* Operating room, *RCT* Randomized controlled trial, *TB* Tuberculosis, *QA* Quality assessment, *QE* Quasi-experimental study

^a^Median age

^b^Age of all participants

^c^Duration refers to the number of minutes per session, and frequency refers to the total number of sessions

^d^Total period of the intervention

Participants were patients (*n* = 11) with community-acquired pneumonia, diabetes mellitus (DM), beta-thalassemia, hypertension, tuberculosis, inflammatory arthritis, and coronary artery disease, parents of asthmatic children (*n* = 1), students (*n* = 6), healthcare providers (*n* = 3) such as nurses and midwives, and other community-dwelling participants (*n* = 5) such as military spouses, older adults, menopausal women, pig farmers, and low-income young adults. The mean age of participants was 42.7 (range, 18.3-68.0) years.

In terms of intervention, twenty-five of the 26 studies in the systematic review provided educational programs as the main intervention, except for one study that used group activity for behavioral modification. The intervention topics included the following: symptom or disease management (e.g., menopause, diabetes, hypertension, and asthma [*n* = 10]), health behavior promotion (e.g., diet, physical activity, sleep [*n* = 10]), psychological health (for example suicide and stress [*n* = 3]), improvement in quality of life (*n* = 2), and dental examination techniques (*n* = 1). Various teaching methods such as lectures (*n* = 5), one-on-one education (*n* = 4), group education (*n* = 11), online education (*n* = 3), and distribution of materials (*n* = 13) were used. Most studies (*n* = 20) used more than two teaching methods. Additionally, most of the intervention providers were healthcare professionals such as doctors, nurses, or health educators. The duration of the intervention per session ranged from 20 to 240 min, and the intervention frequency ranged from 1 to 21. Twenty-one studies (80.8%) included control groups, 11 studies (42.3%) provided usual care such as education, with training programs originally offered. The total sample sizes of the intervention and control groups varied, ranging from 32 to 1,639.

For outcome, we reviewed knowledge as a primary outcome and secondary outcomes (e.g., health-related behaviors, cognitive outcome, quality of life, and physiological outcome). Eleven of the 26 studies in the systematic review reported the knowledge as a primary outcome of the intervention. The review showed studies that measured health-related behaviors (*n* = 11), cognitive outcomes (*n* = 5), quality of life (*n* = 4), physiological outcomes (*n* = 3), and others (*n* = 3). Eleven articles reported that an intervention using the PRECEDE-PROCEED model effectively improved health-related behaviors [7, 17, 19, 20, 25, 26, 29, 30, 32, 38, 42]. Five studies showed that intervention was useful for cognitive outcomes (awareness, attitude, competency, self-efficacy, and self-acceptance) [21–23, 33, 34, 36]. Four studies reported increased quality of life as a primary intervention outcome for patients with DM, migrant tuberculosis, coronary artery bypass grafts, and older adults [18, 31, 35, 41]. Three studies focused on physiological outcomes after interventions such as systolic blood pressure reduction [24], decreased hemoglobin A1c (HbA1c) levels [28], and reduction of musculoskeletal symptoms [39]. The remaining studies found a reduction in stress [27], reduced incidence of pork infected with cysticercosis [37], and improved family empowerment [40].

Additionally, the application of the PRECEDE-PROCEED model was divided into three categories: (1) planning, including the program design; (2) implementation; and (3) evaluation. In 16 studies (61.5%), the PRECEDE-PROCEED model was used for all three categories (planning, implementation, and evaluation). Eight studies (30.8%) applied it only for planning, while the others applied it for planning and implementation (*n* = 2, 7.7%).

### Quality of included studies

We evaluated the methodological quality of all the included studies using the MMAT (Table 2). Of the 26 studies, 7 (26.9%) met all five criteria, and 12 (46.2%) met three or four of the five criteria. Additionally, 7 studies (26.9%) met one or two of the five criteria.

**Table 2:** Quality assessment

Author	Study design	Q1	Q2	Q3	Q4	Q5	MMAT^a^
Adamuz et al. [17]	RCT	Yes	Yes	Yes	Can’t tell	Yes	4/5
Azar et al. [18]	QE	Yes	Yes	Yes	Yes	Yes	5/5
Barasheh et al. [19]	QE	Yes	Yes	Can’t tell	Yes	Can’t tell	3/5
Bazpour et al. [20]	RCT	Can’t tell	Yes	Can’t tell	Yes	Can’t tell	2/5
Bridges et al. [21]	QE	Yes	Yes	Yes	Can’t tell	Yes	4/5
Cannick et al. [22, 23]	RCT	Yes	No	Yes	Yes	Yes	4/5
Chabot et al. [24]	QE	Yes	Yes	Yes	Yes	Yes	5/5
Chiang et al. [25]	RCT	No	Yes	Yes	Can’t tell	No	2/5
Cole & Horacek [26]	RCT	Can’t tell	Yes	No	Can’t tell	No	1/5
Didehvar et al. [27]	QE	Yes	Yes	Can’t tell	Yes	Can’t tell	3/5
Gary et al. [28]	RCT	Yes	Yes	Yes	Yes	No	4/5
Kaewchin et al. [29]	RCT	Can’t tell	Yes	Yes	Can’t tell	Yes	3/5
Kattelmann et al. [30]	RCT	Yes	Yes	No	Can’t tell	No	2/5
Khortwong & Kaewkungwal [31]	QE	Can’t tell	Yes	Yes	Can’t tell	No	2/5
Kim et al. [32]	QE	Yes	Yes	Yes	Yes	Yes	5/5
Lacaille, et al. [33]	QE	Yes	Yes	Yes	Yes	Yes	5/5
Lin et al. [34]	QE	Yes	Yes	Yes	Yes	Yes	5/5
Mazloomymahmoodabad et al. [35]	QE	Can’t tell	Yes	Yes	Yes	Yes	4/5
Moshki et al. [7]	QE	Yes	Yes	Yes	Yes	Yes	5/5
Moshki et al. [36]	RCT	Yes	Can’t tell	Yes	Can’t tell	Yes	3/5
Ngowi et al. [37]	RCT	Can’t tell	Yes	No	Can’t tell	No	1/5
Ranjbaran et al. [38]	RCT	Yes	Yes	Yes	Can’t tell	Yes	4/5
Sezgin, & Esin [39]	QE	Yes	Yes	Yes	Yes	Yes	5/5
Syakurah et al. [40]	QE	Can’t tell	Yes	Yes	Can’t tell	Yes	3/5
Pournaghash-Tehrani & Etemadi [41]	RCT	Can’t tell	Yes	Can’t tell	Can’t tell	Can’t tell	1/5
Walsh et al. [42]	QE	Yes	Yes	No	Yes	No	3/5

*MMAT* Mixed Methods Appraisal Tool, *RCT* randomized controlled trial, *QE* quasi-experimental study

^a^Number of yes responses among the five assessment items

### Effect of intervention

The most common intervention utilizing the theoretical framework of the PRECEDE-PROCEED model was “educational programs.” The educational assessment of the PRECEDE-PROCEED model is classified into predisposing, reinforcing, and enabling factors that affect behavior and environmental changes [4]. Predisposing factors (knowledge, attitudes, beliefs, personal preferences, existing skills, and self-efficacy beliefs) are the basis and motivating precedent for behaviors affecting health and quality of life [3]. In this study, the primary outcome measure, knowledge was included in the meta-analysis. Of the 26 studies, only 11 studies presenting statistical values of educational programs on knowledge were entered into the CMA program. However, the secondary outcome measures were too diverse, so other studies not presented statistical values of the effect of the education program had excluded.

**Fig. 2:**
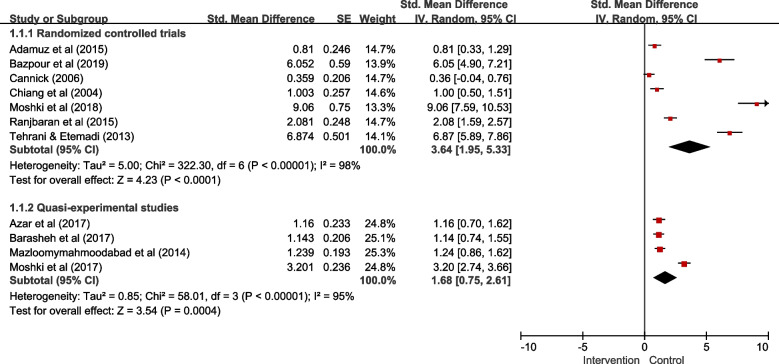
Knowledge (predisposing factors)

Random effects models were developed, and *I*^2^ statistics were used to examine the heterogeneity. The analysis of effect sizes was conducted on seven randomized controlled trials and four quasi-experimental studies, dividing subgroups in accordance with the study design (Fig. 2). For seven randomized controlled trials, the effect size was 3.64 (95% CI 1.95 to 5.33), which was statistically significant (*p* < 0.001). However, the mean effect size was statistically heterogeneous (*Q* = 5.00, *p* < 0.001, *I*^2^ = 98%). The effect size of four quasi-experimental studies was 1.68 (95% CI 0.75 to 2.61), which was also statistically significant (*p* < 0.001) and heterogeneous (*Q* = 0.85, *p* < 0.001, *I*^2^ = 95%). These results indicate that the intervention using the PRECEDE-PROCEED model had statistically significant effects on knowledge changes in both randomized controlled trials and quasi-experimental studies.

**Fig. 3:**
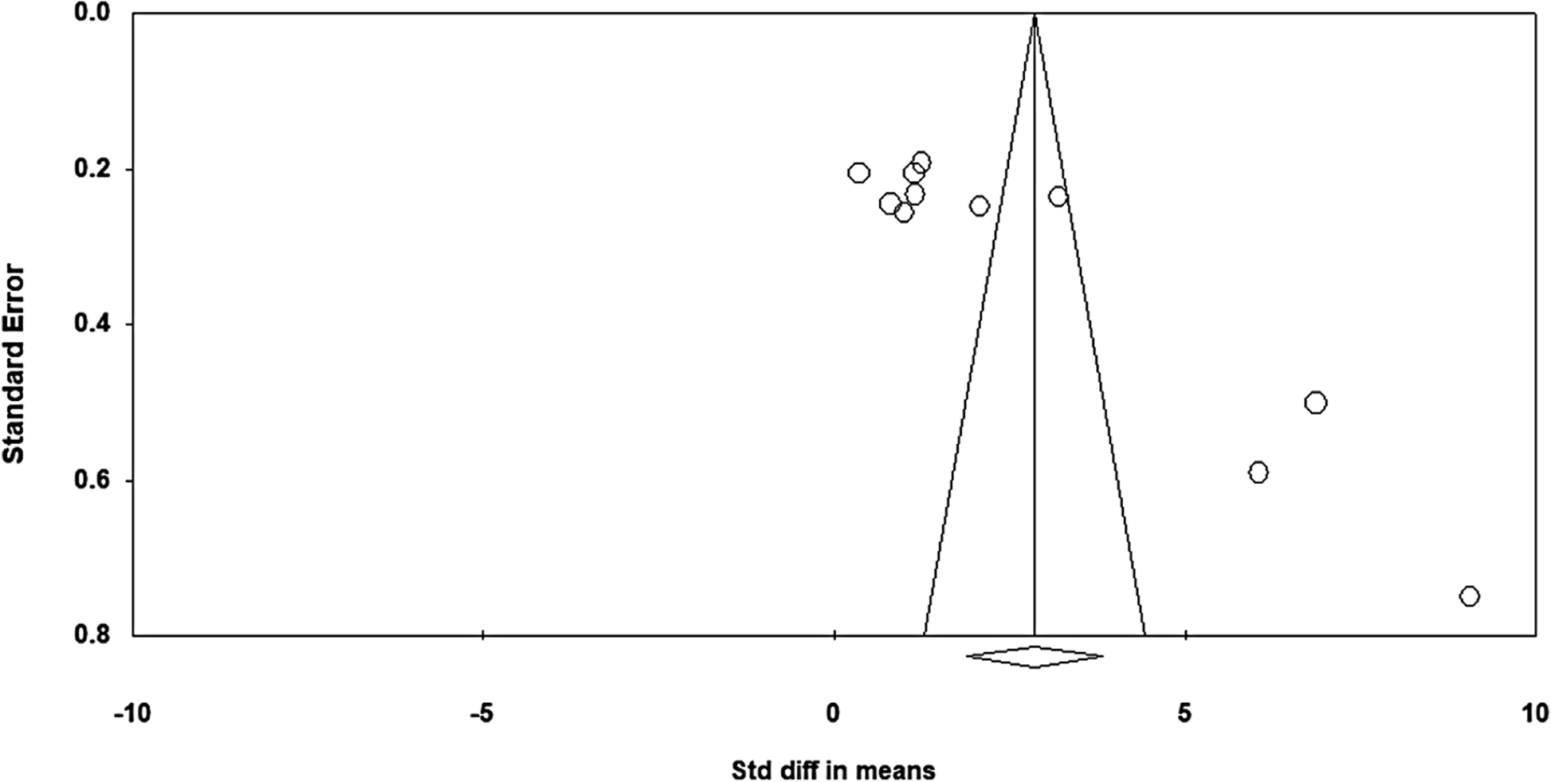
Funnel plot (observed values)

**Fig. 4:**
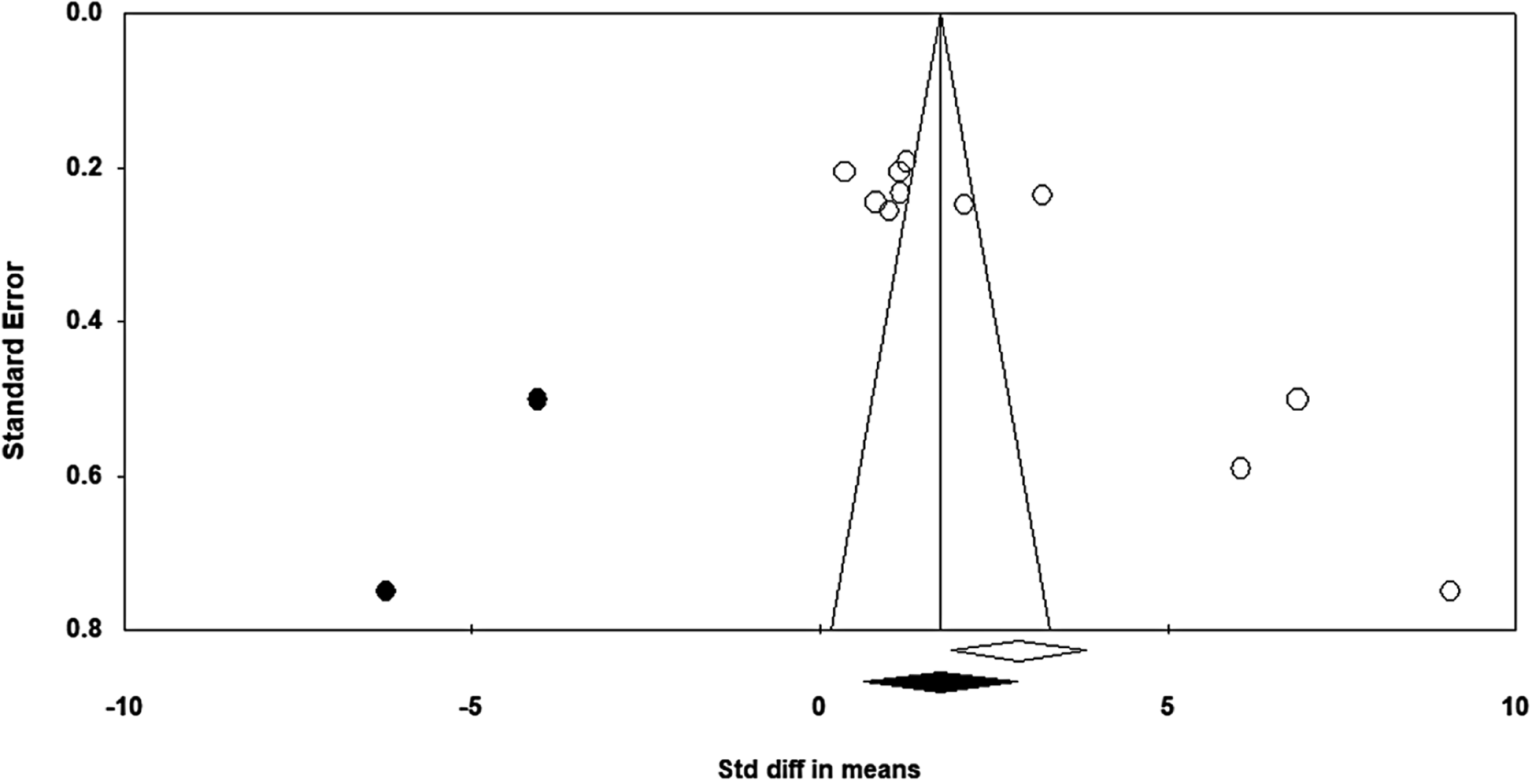
Adjusted funnel plot

This study used a funnel plot to visualize publication bias and asymmetry. The shape of the funnel plot was asymmetric, indicating the presence of publication bias (Fig. 3). Accordingly, trim-and-fill analyses were used to adjust for publication bias. As a result, two possible missing studies were included on the left side of the distribution (Fig. 4). The adjusted mean effect size was calculated as 1.73, which was reduced from the observed effect size of 2.58. However, even after adjusting for publication bias, the mean effect size was still statistically significant (95% CI = 0.63, 2.83). Therefore, it could be interpreted that publication bias did not affect the overall findings of this study and that the PRECEDE-PROCEED model had significant effects on changes in knowledge.

## Discussion

The PRECEDE-PROCEED model is mainly used to guide health promotion strategies in different population groups, with the advantage of applying theoretical frameworks, interventions, and evaluations [4]. In our systematic review, we summarized the characteristics of studies that used the PRECEDE-PROCEED model on health programs. Among the 26 studies, 50% were published from 2015 to 2020, and 34.6% (*n* = 9) were conducted in Iran. This might be related to an increased interest in modifying health behaviors and healthy lifestyles in Iran [18, 20]. In particular, the importance of health education for behavioral changes towards healthy lifestyles is increasing in Iran; health education is being implemented by applying the PRECEDE-PROCEED model for health programs in different population target groups [8, 43], and its effectiveness has been proven in several studies [8, 43]. Additionally, the study participants in these previous studies varied widely, including patients with health problems and community-dwelling healthy people such as students, medical staff, and the general public comprising adolescents and older adults. Therefore, this theory provides education across all ages for a population group. With respect to the application of the PRECEDE-PROCEED model, most studies applied the model for planning, implementation, and evaluations. However, 38.5% (*n* = 10) of the studies partially applied the model to program planning and implementation. This theory could be utilized as a roadmap that provides a structure for systematically planning and evaluating health behavior change programs and presents specific directions for changing behavioral programs [3]. Therefore, a series of processes are systematically applied to a health program through a theoretical framework.

Most studies used education as an intervention method. The PRECEDE-PROCEED model allows for evidence-based best practices in health promotion intervention [4]. The model uses an appropriate educational approach because it conducts population-centered interventions and provides practical guidelines for health programs [4, 44]. Two or more methods have been used for most educational interventions, with group education and distribution of materials being the most common. Group education, including group activity and discussion, is useful in sharing experiences, encouraging others [25], and increasing interactions among people [26]. The group education method would, in theory, be an effective way to improve mutual understanding through discussion. The distribution of materials is an appropriate educational method to pass knowledge to the entire population simultaneously [37]. Through leaflets, booklets, and posters, it is possible to deliver the general overview or the main contents of a message to the target audience, and because it is summarized in pictures rather than texts, even those who cannot read the text can understand it. Thus, leaflets, booklets, and posters are useful reference materials [37].

In the analyzed studies, the main intervention was symptom or disease management and promoting healthy behavior such as diet management and active physical activity. Interventions utilized predisposing, reinforcing, and enabling factors based on educational and ecological assessment, one of the model’s components. Symptoms or disease management intervention mainly utilized predisposing and reinforcing factors. Predisposing factors were knowledge, complications, and management methods about a specific disease; in contrast, reinforcing factors were customized management through experts [19] or family and friend support about disease management [31]. These interventions changed behaviors by improving understanding of the disease and self-management ability [33]. Interventions about promoting healthy behaviors utilized various methods such as educational messages and short message services (SMS), distribution of compact discs (CDs), posting materials [27], and distribution of emails with videos and web portals [30]. This not only changed participants’ behaviors [27] but also allowed them to enjoy the program by setting goals [30]. It is helpful to use the appropriate methods by mobilizing various resources to promote healthy behaviors. In addition, the secondary outcomes of this study were mostly health-related behaviors. This might mainly be because behavioral changes as outcome variables were evaluated based on health-related interventions and educational programs that applied the model. Therefore, considering the characteristics of this model, it is appropriate to use health-related behaviors as an outcome variable.

Although a systematic review was published in 2020, a meta-analysis has not yet been performed. A recent systematic review of 26 studies utilizing the PRECEDE-PROCEED model as a screening tool reported that this model could provide a framework that enhances understanding and knowledge [45]. In addition to a systematic literature review, this study performed a meta-analysis to evaluate the effectiveness of the PRECEDE-PROCEED model. Our meta-analysis results showed that intervention programs applying the PRECEDE-PROCEED model effectively improved the knowledge of predisposing factors in educational and ecological assessment. Predisposing factors are antecedents that motivate a particular behavior [3, 45]. This suggests that people with high health knowledge may be more likely to engage in health-related behaviors.

This study has some limitations. First, we only included studies published in English. Second, since studies applying the PRECEDE-PROCEED model were significantly diverse regarding their study design and outcome measures and data are not available for most of the specified outcome measures, it is difficult to calculate the effect sizes. We tried to overcome this limitation by including knowledge as the primary outcome using meta-analysis. However, since only knowledge among the proposing factors was included in the meta-analysis, there are limitations regarding how the effects should be interpreted. Despite these limitations, our study has several strengths. The PRECEDE-PROCEED model could be a useful theoretical model for designing health programs. Overall, this study provided an objective assessment of the effects of interventions that applied the PRECEDE-PROCEED model on knowledge enhancement.

## Conclusions

Our systematic literature review and meta-analysis showed that interventions using the PRECEDE-PROCEED model mainly used education methods and measured health-related behaviors. This model is a useful tool across populations among all age groups. Specifically, knowledge was effectively improved when the intervention was conducted using this model. In the future, it will be useful to use the PRECEDE-PROCEED model when implementing programs for disease management and health promotion. 

## Data Availability

Not applicable.

## Appendix

Table 3

**Table 3:** Search strategy

Database	Search terms
PubMed	(Precede-Proceed [All Fields] AND (model [All Fields] OR health promotion [All Fields] OR health promotion [MeSH Terms] community health planning [All Fields] OR community health planning [MeSH Terms] OR population based planning [All Fields] OR health program [All Fields] OR program evaluation [All Fields] OR program evaluation [MeSH Terms] OR intervention [All Fields])) OR (Precede Proceed Model [All Fields]) OR (Precede Proceed [All Fields])

## Abbreviations

PRECEDE-PROCEED: Predisposing, Reinforcing, and Enabling Constructs in Educational Diagnosis and Evaluation-Policy, Regulatory, and Organizational Constructs in Educational and Environmental Development
PRISMA: Preferred Reporting Items for Systematic reviews and Meta-Analyses statement
CINAHL: Cumulative Index to Nursing and Allied Health Literature
RISS: Regional Information Sharing Systems
MMAT: Mixed Methods Appraisal Tool
CMA: Comprehensive Meta-Analysis
DM: Diabetes Mellitus
HbA1c: Hemoglobin A1c
SMS: Short Message Service
CD: Compact Disc
